# Attention and residual mechanism-based CNN architecture (ARC-Net) with enhanced fairness generalization for deepfake facial image detection

**DOI:** 10.1371/journal.pone.0340099

**Published:** 2026-04-06

**Authors:** Md Shihab Reza, Farhana Elias, Monirul Islam Mahmud, Nova Ahmed

**Affiliations:** 1 Department of Electrical and Computer Engineering, North South University, Dhaka, Bangladesh; 2 Design Inclusion & Access Lab, North South University, Dhaka, Bangladesh; Karabük Üniversitesi: Karabuk Universitesi, TÜRKIYE

## Abstract

Deepfake (DF) content poses a major challenge to digital media authentication, that can mimic facial movements, creating realistic replicas that risk spreading misinformation and enabling harassment, as can be seen in Bangladesh. Previous studies applied residual convolutional blocks or attention mechanisms for DF detection; however, these approaches often treat these components in isolation and lack explicit consideration of fairness, out-of-distribution generalization, statistical analysis or artifact-focused detection. Our study introduces an approach, called ARC-Net, which uses a combination of attention and residual convolutional layers along with the EfficientNet B0 base, the attention mechanism of which allows the model to pay more attention to details that could resemble the ones seen in non-perfectly executed DF, which enhances the model’s potential to distinguish them, addressing limitations in pre-trained and also advanced DL models. A dataset of 500 real images from Bangladesh combined with a 140k real and fake faces dataset was used to train and test our model alongside four pre-trained DL models. ARC-Net performed much better than the other traditional and state of art methods with 99% accuracy, 1.0 precision, 0.97 recall and 0.98 F1 score, reaching the highest level of reliability in spotting DF images. To assess the external reliability and generalizability of ARC-Net, the model was evaluated on the Deepfake Dataset and the Deepfake Database datasets, achieving consistently high and balanced performance across different scales. Three out-of-distribution (OOD) experiments were conducted, where the first one evaluated South Asian images, showing that incorporating fewer than one percent real Bangladeshi images reduced the false positive rate by more than half and improved probability calibration, while the remaining two cross-dataset experiments demonstrated strong transferability. An ablation study further showed the impact of different components within a model by systematically removing or modifying them and statistical significance between competing classifiers was assessed using McNemar’s Statistical test. In addition, we’ve applied Explainable AI (XAI) techniques, Grad-CAM and LIME to offer transparency of the results, as giving attention to the facial region is also important for DF detection. This study will help advance DF detection by integrating ARC-Net’s attention-residual mechanisms and XAI, offering insights for developing models in security and media forensics.

## 1 Introduction

One notable advancement in recent years is the emergence of deepfakes (DF), referred to as intelligence generated content that appears authentic but is actually fake [4]. Generating DF facial images and videos has become easier and realistic because of the advancement of DF technology. Generative adversarial networks (GANs) introduced by Goodfellow et al [5] in 2014, was recognized as one of the algorithms for generating DF content. Though DF can offer advantages in fields such as education, art, activism, and self-expression, they also pose risks of being exploited for generating material, spreading narratives, and provoking negative emotions in individuals. Due to the availability of technologies people are now sharing more data than ever before such as in social media as it leads to availability of a large number of videos and images. In regions like Bangladesh and other countries in South Asia, manipulated facial images are primarily utilized to produce content, circulate misleading information, and instill feelings of fear or disgust among the people. Because of the way AI is misused to target people with harassment, thousands of women have had to take down their pictures and videos from social media sites. Also, this deepfake technology is also used to increasingly attack female politicians in South Asia [6] and [7]. DF images lead to serious harm, especially impacting marginalized communities, like people with disabilities and youth as individuals, in the LGBTQ+ community, and racial minorities; both men and women go through it too but men tend to report higher instances of being victims or perpetrators of DF [8].

Most of the DF facial detection techniques rely on feature selection and DL techniques. The research community has been actively proposing different approaches and solutions for detecting DF images and videos, trying to address these issues posed by DF media. Several traditional DL models such as Xception, NAS Net, MobileNet, VGG16, EfficientNetV2, VGG19, Vision Transformers (ViTs), proGAN, SAGAN, DCGAN and many more are also utilized for detecting DF images or videos in [9–11], and [12]. Several hybrid frameworks like MMGANGuard, FF-LBPH DBN and many more are proposed for DF detection [13], and [14]. Apart from DL techniques some blockchain-based federated learning (FL) methods also utilized for secured Deepfake system [15] and XAI techniques mainly LRP and LIME utilized to provide representations of the model’s attention, on important image areas [16].

Deepfake studies advances rapidly and prior research works have already applied attention and residual mechanisms, but most of the works have limitations. Limitation includes a less usage of diverse datasets and resource constraints which limit the ability to develop innovative deepfake solutions, sometimes models may achieve high accuracy, but get focused by non-relevant image content, such as the background, or focus on regions that are not critical for detecting DF facial images. Lastly, most existing deepfake detection studies are limited to single-dataset evaluation such as The 140k real and fake faces, FaceForensics++, CelebDFv2 datasets [16–27] and lack systematic OOD testing, ablation analysis, statistical test and explainability in a single work. To the best of our knowledge, this is the first work that jointly addresses OOD evaluation, ablation analysis, statistical analysis and explainability of deepfake detection with robust generalization and transparent model insights. The contributions of this study are summarized as follows:

- Proposed ARC-Net, a framework integrating attention and residual convolutional layers with an EfficientNetB0 backbone, which enables accurate capture of fine-grained features in imperfectly generated deepfakes.
- Extended the 140k real-and-fake faces dataset [1] with 500 real images from South Asian individuals in Bangladesh, introducing regional diversity and improving subgroup performance. This inclusion reduced the false positive rate from 22.0% to 10.0% and improved Brier score from 0.180 to 0.110.
- We analyzed robust generalization, statistical testing and out-of-distribution (OOD) evaluation, where ARC-Net achieved 99.0% accuracy on the 140k Real/Fake Faces dataset [1] with added Bangladeshi images, 97.6% on the Deepfake Dataset [2], and 99.3% on the Deepfake Database [3], with balanced precision, recall, and F1-scores. Also, cross-dataset evaluations further extended with 3 different out-of-distribution (OOD) evaluations. Another key aspect, Ablation studies revealed that combining residual and attention modules improved accuracy by 5.0% over the baseline. Finally, statistical analysis using the McNemar’s test yielded a p-value of 0.028, indicating statistically significant results on 100 held-out real South Asian images.
- Lastly, we enhanced model explainability through Gradient-weighted Class Activation Mapping (Grad-CAM) and Local Interpretable Model-agnostic Explanations (LIME) to correctly focus on relevant facial regions such as eyes, mouth, and texture details when distinguishing between real and fake images.

The remaining sections of the paper are structured as follows; Section 2 presents an overview of the background study while Section 3 details the materials and methods utilized in the research process. Subsequently, in Section 4 is the analysis of the results and Section 5 concludes by summarizing the findings, and discusses the impact on the field as well as offering insights, for future research directions.

## 2 Related works

Hitherto, numerous researchers have dedicated their efforts to developing techniques for distinguishing deepfake facial images and videos in recent years. Several relevant studies have been reviewed with key contributions from various researchers are discussed below.

Zhang et al. [28] used learning techniques to optimize DF detection, and while testing across many models and training on photos from a single DF model, they achieved an accuracy rate of 97.04%. Ismail İlhan et al. [19] implemented the NASNetLarge CNN to effectively recognize images, achieving a precision rate of 96.7%. Research conducted by Atwan et al. [11] proposes a framework utilizing convolutional neural networks (CNNs) and deep transfer learning techniques to distinguish between real and DF photos. Suganthi ST et al. [14] implemented the FF-LBPH DBN technique for detecting DF images and this technique achieved 98.82% accuracy on the CASIA-WebFace dataset and 97.82% on the DFFD dataset but its generalization to more complex datasets remain to be explored. Thipparthi Vignesh et al. [29] CRNet, a new type of residual network which utilizes Convolutional Long Short-Term Memory (LSTM) designed to interpret sequences of images from a video. In study [30] implements machine and DL methods on a dataset of DF and authentic videos to identify manipulations and results showed that CNN model achieves 94% accuracy, while the VGG model reaches 88% accuracy. Arpita Dharet al. [31] used a network model that reached an accuracy rate of 98.77% in distinguishing between images and DF ones, outperforming methods. In study [18], researchers conducted an analysis of DL models, where the findings indicated that the tuned VGG16 model achieved 90% accuracy, 91% precision, high recall rates and significant F1 scores. In research conducted in [9] the issue of risks is tackled through the utilization of four network methods: Xception, NAS Net, MobileNet and VGG16. Their Deep Fake Predictor (DFP) attained an accuracy rate of 94% by integrating VGG16 and convolutional neural networks. In study [32] involved creating a dataset of 83,000 images—half genuine and half DF from nine GAN architectures and four Diffusion Models and developed a hierarchical detection approach: the first level classifies real versus AI-generated images, the second distinguishes between GANs and DMs, and the third identifies specific architectures. Their experiments achieved over 97% accuracy, surpassing state-of-the-art methods.

Transfer learning is used to improve the accuracy of DF models, specifically comparing different versions of the EfficientNetV2 architecture, in a study conducted by Harsh Vajpayee et al. [10]. Though the robustness of the method on compressed images should be tested further. Using transfer learning, Md.Tahmid Hasan Fuad et al. [20] present proposed their DL approach which is built on CNN and Wide ResNet architectures, outperforms other pretrained models with an accuracy of 82.4% on the DF Challenge dataset of 3,762 films and 83.47% on a newly constructed dataset of 121 videos. Despite its moderate performance, the model shows promise, though more advanced datasets should be used to test its generalizability. Shahzeb Naeem et al. [21] conducted a research study on image features including ANOVA testing and findings suggest that DL algorithms perform well in recognizing images using the ViT Patch 16 model showcasing performance metrics: sensitivity of 97.37% specificity of 98.69%, precision of 97.48% and accuracy of 98.25%. Davide Alessandro Coccomini et al. [33] uses Vision Transformers with EfficientNetV2 using the ForgeryNet dataset in a cross-forgery context and their finding suggest that while EfficientNetV2 tends to perform better with familiar training methods, Vision Transformers are better at generalizing. Muhammad Asad Arshed et al. [12] evaluated Vision Transformers (ViTs) for multiclass DF detection. A hybrid DL model with the combination of Generative Adversarial Networks (GANs) and Residual Neural Network (RESNET) build for detecting fake faces in study [34]. In study [35] proposed a solution that employs a stacking-based ensemble approach that combines features from two well-known DL models, Xception and EfficientNet-B7. In experiments, the model achieved 96.3% accuracy on the Celeb-DF (V2) dataset and 98.00%on the FaceForensics++ dataset. In study [36] presents a hybrid approach combining Convolutional Neural Networks (CNN) and Deep Convolutional Generative Adversarial Networks (DCGANs) to detect and eliminate DF multimedia content. In a study by [13], MMGANGuard was introduced as a system that combines Gram Net, ResNet50V2 and DenseNet201, through transfer learning and reached an accuracy rate exceeding 97% in detecting DF videos within the StyleGAN dataset. In study [37], the authors propose a new detection method that combines deep neural networks with detailed artifact features to improve adaptability for different types of facial synthesis. In study [38] compared supervised and self-supervised DL models for DF detection across four different datasets: FakeAVCeleb, CelebDF-V2, DFDC, and FaceForensics++ and their findings indicate that MViT-V2 and Res2Net-101, consistently outperformed CNNs, particularly in cross-dataset scenarios, while requiring fewer parameters. Self-supervised transformers, such as ViT-Base with DINO, showed better generalization than supervised models. Image augmentations further improved transformer performance but had less impact on CNNs. A new approach is introduced in study [17] by using a Support Vector Machine (SVM) classifier to identify fake human faces; it compares two detection methods such as Principal Component Analysis (PCA) and SVM without PCA and findings showed that with the SVM model using PCA achieving an impressive accuracy of 96.8%, while the standard SVM model reached only 72.2%. Badhrinarayan Malolan et al. [16] in their study trained a Convolutional Neural Network on a dataset taken from FaceForensics DF Detection Dataset and they applied XAI methods, like LRP and LIME to provide representations of the model’s attention, on important image areas. Heidari et al. [15] proposed a blockchain-based federated learning (FL) solution for DF detection that preserves data source anonymity which combines SegCaps and convolutional neural networks (CNNs); This approach demonstrates the potential of federated learning, though its implementation in practical, large-scale settings needs further exploration. In study [39], introduced the Secure DF Detection Network (SecDFDNet) and results show that SecDFDNet can detect DF faces without revealing private input, achieving accuracy similar to the plaintext DFDNet while outperforming other models.

E. Şafak et al. [23] used a stacking ensemble learning method consisting of a lightweight convolutional neural network to detect real and fake face images, achieving a 96.44% accuracy by combining MobileNet and MobileNetV2 with an optimised EfficientNetB0 model. Kothandaraman D et al. [24] fine-tuned a pretrained InceptionResNetV1 model on the VGGFace2 dataset for real or fake facial image classification with 97% accuracy on the training set and on the testing set. Jatin Sharma et al. [25] proposed a CNN-based model that utilises both generative adversarial networks and data augmentation to classify real and fake face images with accuracy up to 95.85% on single datasets. The accuracy achieved was only further increased to 98.79%, 75.79%, and 95.52% on three benchmark datasets through ensembling pretrained VGG16 and ResNet50 models, respectively, outperforming previous state-of-the-art approaches. TruceNet [26] is a CNN-based model that reached a 94.2% accuracy in the DF detection by relying on multiple features of images and outperforming most state-of-the-art solutions. H. Alshammari et al. [27] fine-tuned the MesoNet model to detect DF, improving its accuracy from 87.1% to 96.2% on a dataset of 140K images. Liwei Deng et al. [40] proposed a multi-label classification approach for DF detection based on a detail-enhancing attention module and global-local transformer decoder. JunShuai Zheng et al. [41] proposed a DF detection model for embodied AI based on balanced contrastive learning and a multi-scale attention interaction module. TSFF-Net [42] improves deepfake video detection by combining spatial and frequency domain features. It achieves high accuracy (97.7%–98.9%) in detecting DF, even in low-quality videos. Mathews et al. [43] showed MesoInception-Net achieved 99.87% validation accuracy on DFIM-HQ dataset while exploring Grad-Cam based visualization. Furthermore, B. Sugiantoro [44] explored ResNet152V2 + Grad-CAM model to get the Highest Precision of 90% for fake and 92% for real images in the FFHQ dataset with explainable AI technique Grad-Cam.

After a thorough review of previous works, it has been observed that most of the DF detection studies exhibit certain research gaps. A lot of approaches are sensitive to dataset biases and are not very robust to varied or low-quality data. Furthermore, some techniques are based on advanced DL approaches, but only a few of them consider the interpretability of the model decisions, and many models show dispersed attention patterns that make it difficult to identify the crucial regions that contain the key DF artefacts. Motivated by these challenges, this work introduces ARC-Net, a residual–attention enhanced architecture built on EfficientNetB0 and trained on a hybrid dataset to mitigate geographical bias. Beyond strong baseline performance, we provide a comprehensive evaluation pipeline such as ablation studies to quantify component contributions, McNemar’s test to establish statistical significance, and extensive out-of-distribution experiments to assess generalization. Furthermore, we integrated Grad-CAM and LIME explainability techniques, validating the model’s focus on important facial regions.

## 3 Materials and methods

**Fig 1:**
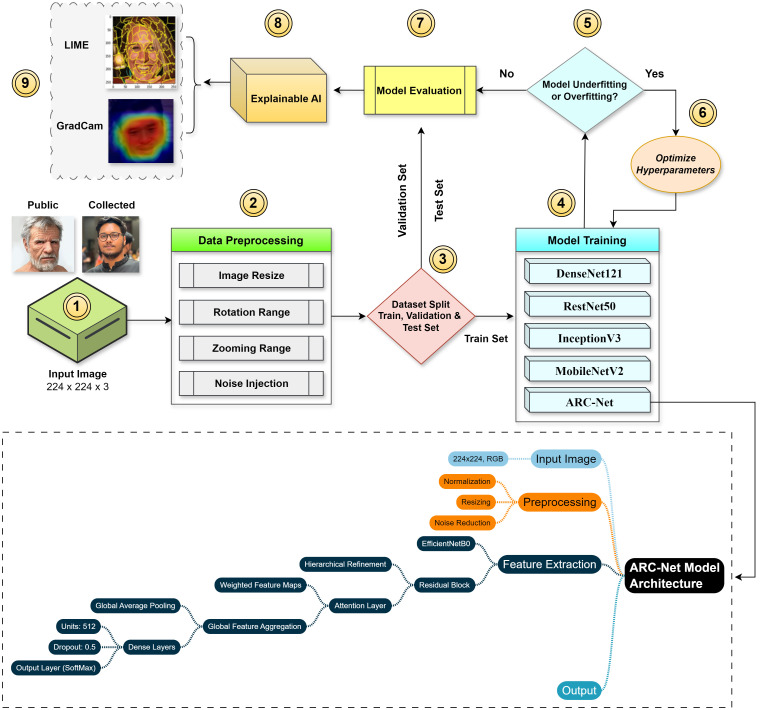
Block diagram of the proposed ARC-Net framework integrating residual and attention modules for deepfake image detection.

Our method for predicting the authenticity of an image involves utilizing a deep convolutional neural network. The framework is formulated as a binary classification task with two classes, Real and Fake. We have evaluated the training accuracy, testing accuracy, precision, recall, F1 score and AUC performance of our suggested models for result evaluation. Our overall research workflow is shown in Fig 1.

### 3.1 Dataset details

**Fig 2:**
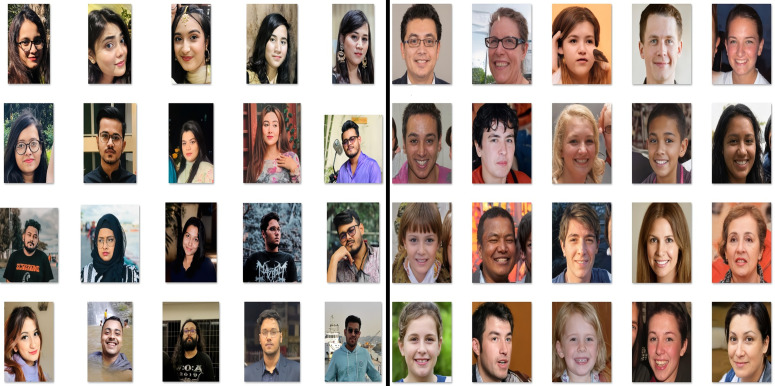
Sample images of extended dataset: **(a)** Real Images and **(b)** Fake Images.

This research uses a combination of data sources, merging images of 140k real and fake faces dataset [1], with 500 collected images from our side. The 500 images we collected included real facial images of Bangladeshi people of different genders and ages. During data collection, written informed consent was obtained from all participants. They were explicitly informed that the data would be used solely for research purposes, that the study involved no risk, and that confidentiality and data security would be maintained. The data collection process was conducted from 6th January 2025–4th April 2025, approved by the Institutional Review Board (IRB)/Ethics Review Committee (ERC) of North South University, Bangladesh (Application No. 2024/OR-NSU/IRB/1109). The individual in this manuscript has given informed written consent (as outlined in the PLOS consent form) to publish these case details. As all face images represent adults and no minors or vulnerable individuals were included, so, informed consent from guardians was not required. For Generalizability Check of the proposed ARC-Net approach the Deepfake Dataset [2] and Deepfake Database [3] are used. All datasets are divided into training, validation, and test sets. The 140k real and fake faces dataset comprises a total of 140,000 images, including 70,000 normal human faces and an equal number of showing deep faked human faces. Among the images, 70,000 were taken from Flickr Faces HQ (FFHQ) [1], a high-quality image dataset specifically tailored for adversarial networks (GAN). The training set contains 100,000 images, the validation set 20,000, and the test set contains 20,000 overall real and fake face images. We added our collected 200 face images into each of the Training set and Validation real image section and the rest of the 100 collected images into the Test data real image section. The Deepfake Dataset [2] contains 102,100 images in its training set and 20,000 images in both its validation and test sets. The Deepfake Database [3] contains 12,023 images in its training set, 7,104 images in its validation set and 330 images in its test set. Table 1 shows the datasets distribution for the training, validation, and test sets.shows the dataset distribution for the training, validation, and test sets. Figure 2 present samples of real collected images and fake images from hybrid datasets.

**Table 1:** Dataset split details for DeepFake datasets used in ARC-Net evaluation.

Description	140k Real/Fake Faces [1]	Deepfake Dataset [2]	Deepfake Database [3]	Purpose
**Train Set**	100,200	102,100	12,023	Used for model training
**Validation Set**	20,200	20,000	7,104	Used for performance monitoring
**Test Set**	20,100	20,000	330	Used for final model evaluation
**Total**	140,500	142,100	19,457	

### 3.2 Data preprocessing

Data preprocessing plays a critical role in enhancing input image data quality, mitigating overfitting, and improving model generalization. To improve the generalization ability of the model and normalize the input images, massive data preprocessing was carried out in this study. As seen in Table 2, all datasets were preprocessed with a well-defined and standardized preprocessing pipeline to ensure reproducibility. Each input image was first resized to 224 × 224 × 3 to ensure compatibility with backbone networks. Input images were resized using the high-quality Lanczos interpolation method. Normalization was performed by rescaling pixel values to the range [0,1]. To increase robustness and reduce overfitting, standard data augmentation techniques were applied: random horizontal flipping (probability = 0.5), random rotation up to ±12°, and random zooming with a scaling factor of up to 1.1. Additionally, Gaussian noise with standard deviation σ=0.03 was injected to mimic sensor-level distortions, compression artifacts, and low-light conditions commonly observed in real-world DeepFake images. After noise addition, values were clipped to the [0,1] range to preserve valid pixel intensity distributions. These augmentations were applied during training only, while validation and test images were processed with resizing and normalization only. This noise injection encourages the model to learn more robust facial representations by preventing overfitting to overly clean images, while all pixel values were clipped to remain within the normalized [0,1] range. To enhance the robustness of the training set, images underwent data augmentation, whereas validation and test sets were only rescaled, without any additional transformations, to ensure unbiased performance evaluation. The same preprocessing pipeline—including resizing, rotation, zooming, and Gaussian noise injection were consistently applied across all DeepFake datasets used in this study. By combining these approaches, it helped ensure that the model was trained on a fully preprocessed and realistic dataset, which improved generalization on unseen data.

**Table 2:** Preprocessing and Data Augmentation Techniques with Parameters.

No.	Technique	Parameter Values	Details
1	Image Resize	224 × 224 × 3	Ensures compatibility while balancing feature extraction.
2	Normalization	[0,1] scaling	Standardizes pixel intensities for stable training.
3	Horizontal Flip	*p* = 0.5	Introduces left–right orientation variability.
4	Rotation	±12°	Simulates natural head pose changes.
5	Zoom	up to 1.1×	Adds scale variability for better generalization.
6	Gaussian Noise	σ=0.03, clipping to [0,1]	Creates robustness against adversarial DF.

### 3.3 Models & evaluation

In this study, we used standard backbone architectures of DenseNet-121, MobileNetV2, ResNet-50, and InceptionV3, all initialized with weights pre-trained on the ImageNet dataset. These backbones were employed without architectural modifications, serving as baselines for comparison. In addition, we developed our proposed ARC-Net framework, which incorporates residual and attention mechanisms specifically tailored for DeepFake detection. DenseNet121 is well known for its connections between layers, which help in reusing features and improving the flow of gradients [22]. We started with a DenseNet121 model, excluding the final classification layer to enable customization. In this setup DenseNet121 serves as the feature extractor. The GlobalAveragePooling2D layer condenses each feature map into a value reducing parameters and preventing overfitting. The last layer consists of a neuron with a sigmoid activation function. Training occurs over 30 epochs to strike a balance, between performance and training time. MobileNetV2 strikes a balance between accuracy and efficiency which is essential for handling the vast amounts of data involved in spotting DF [14]. To capture patterns to DF detection two dense layers with ReLU activation functions (1024 and 512 neurons) are incorporated. ResNet50 [32] is an effective image classification model that can be trained on big datasets and produce cutting-edge results. It utilizes pooling, then a dense layer, with ReLU activation batch normalization and a final dense layer with softmax activation for binary classification (distinguishing between DF and real content). To extract features from images, the InceptionV3 [33] architecture employs a number of convolutional, pooling, and inception modules. A layer with 1024 neurons and ReLU activation is included to introduce non- linearity allowing the model to grasp patterns. In addition, the model ran alongside the ReduceLROnPlateau callback, which decreases the learning rate when the validation performance plateaus, as well as early stopping when faced with overfitting, as data preprocessing takes care of this as well. The learning rate is decreased during this process to prevent changes that may interfere with the established weights. Table 3 shows the details of all model parameters.

**Table 3:** Model Parameter details.

Model	Parameters
DenseNet121	Optimizer: Adam; Loss: sparse_categorical_crossentropy; Dense units: 1024; Activation: ReLU, Softmax; Weight init: ImageNet
InceptionV3	Optimizer: Adam; Loss: sparse_categorical_crossentropy; Dense units: 1024; Activation: ReLU, Softmax; Weight init: ImageNet
MobileNetV2	Optimizer: Adam; Loss: sparse_categorical_crossentropy; Dense units: 1024; Activation: ReLU, Softmax; Weight init: ImageNet
VGG16	Optimizer: Adam; Loss: sparse_categorical_crossentropy; Dense units: 1024; Activation: ReLU, Softmax; Weight init: ImageNet
EffecientNetB0	Optimizer: Adam; Loss: binary_crossentropy; Batch size: 32; Activation: Swish, Sigmoid; Weight init: GlorotUniform
ResNet50V2	Optimizer: Adam; Loss: binary_crossentropy; Activation: ReLU, Sigmoid; Weight init: HeNormal (Conv)
ARC-Net	Learning rate: 5e-4; Optimizer: Adam; Batch size: 64; Loss: sparse_categorical_crossentropy; Dropout: 0.5; Dense units: 256; Activation: ReLU, Softmax; Regularization: BatchNormalization; Weight init: ImageNet

### 3.4 Attention mechanism based ARC-Net architecture

We introduce ARC-Net, an attention-aware and spatial-oriented Convolutional Neural Network (CNN) architecture which aims to enhance the feature extraction ability by incorporating not only with advanced residual connection but also attention mechanisms. ARC-Net uses an EfficientNetB0 backbone as its base feature extractor which provides a strong and computationally efficient foundation for transfer learning since it generalizes well while being less resource demanding in comparison to the standard bases. We augment EfficientNetB0 with specialized residual blocks and attention mechanisms, hand-picked for solving various nuances of DF detection such as minor pixel level perceptible differences. We set up ARC-Net using EfficientNetB0, a compact CNN trained on ImageNet for high performant and transferable feature representation in different types of imagery data. Compound scaling allows Scaling the width, depth and resolution at same time so that a trade-off curve between performance of image classification accuracy and computation cost can be balanced in an efficient way. The Pseudo code and symbol notations of proposed ARC-Net approach based on this work has been summarized in Algorithm 1 and Table 4.

**Algorithm 1 Pseudocode of ARC-Net: Attention Residual based CNN for DeepFake Face Detection with XAI Integration**

**Require** $X_{\text{train}},Y_{\text{train}},X_{\text{test}},Y_{\text{test}}$, θ: termination criterion

**Ensure** Pred, Acc

1:   Shuffle $\left(X_{\text{train}},Y_{\text{train}}\right)$ and split into mini-batches $\{(X^{\left(i\right)},Y^{\left(i\right)}){\}}_{i=1}^{m}$

2:   Base←EfficientNetB0(weights=imagenet,include_top=False)

3:   **for** each batch $\left(X^{\left(i\right)},Y^{\left(i\right)}\right)$ **do**

4:     $x\leftarrow Base(X^{\left(i\right)})$

5:     **for** each block *j* **do**

6:      Residual: $y_{j}\leftarrow {ℱ}_{j}(x_{j},\{W_{j}\})+x_{j}$

7:      Attention:

$M_{c}=\sigma ({\text{FC}}_{2}(\delta ({\text{FC}}_{1}(\text{GAP}(y_{j}))))))]$

$M_{s}=\sigma (f^{7\times 7}([\text{AvgPool}(y_{j});\text{MaxPool}(y_{j})]))$

8:      $x_{j+1}\leftarrow M_{s}\cdot (M_{c}\cdot y_{j})$

9:     **end for**

10:     GlobalAveragePooling: *F* = GAP(*x*_*j*+1_)

11:     Dropout + Dense: $\hat{y}=\sigma (W^{T}F+b)$

12:     Update weights by minimizing:

${ℒ}_{\text{BCE}}=-[Y^{\left(i\right)}\log(\hat{y})+(1-Y^{\left(i\right)})\log(1-\hat{y})]$

13: **end for**

14: Fine-tune top-*k* layers with learning rate $\eta =10^{-5}$

15: Prediction: Pred = round(*Net*(*X*_test_))

16: Accuracy: $\text{Acc}=\frac{1}{n}\sum_{i=1}^{n}⊮(Pred_{i}=Y_{\text{test},i})$

17: **return** (Pred, Acc)

18: **Grad-CAM:** For class *c*, compute importance weights for feature map *A*^k^:

$\begin{array}{c} \alpha_{k}^{c}=\frac{1}{Z}\sum_{i}\sum_{j}\frac{\partial y^{c}}{\partial A_{ij}^{k}} \end{array}$

$\begin{array}{c} {\text{GradCAM}}^{c}=\text{ReLU}\left(\sum_{k}\alpha_{k}^{c}A^{k}\right) \end{array}$

19: **LIME:** For an input *x*, generate perturbed samples $x^{\prime }\in 𝒵$ and predict *f*(*x*’):

$\text{LIME}(x)=\arg\min_{g\in G}ℒ(f,g,\pi_{x})+\Omega (g)$

**Table 4:** Notation and Symbols Used in Algorithm 1.

Symbol	Description
$X^{\left(i\right)},Y^{\left(i\right)}$	*i*-th mini-batch of training data (inputs and labels)
*j*	Block index within the CNN backbone
θ	Termination criterion (validation loss threshold)
${ℱ}_{i}(\cdot )$	Residual mapping function with weights
*M*_*c*_	Channel attention map
*M*_*s*_	Spatial attention map
${\text{FC}}_{1},{\text{FC}}_{2}$	Fully connected layers
δ(·)	ReLU activation function: δ(z)=max(0,z)
σ(·)	Sigmoid activation function
$\hat{y}$	Predicted output (probability)
*W*, *b*	Weights and bias of dense layer
${ℒ}_{\text{BCE}}$	Binary Cross-Entropy loss
η	Learning rate during fine-tuning
⊮(·)	Indicator function (1 if condition true, else 0)
*A* ^*k*^	*k*-th feature map in Grad-CAM
$\alpha_{k}^{c}$	Grad-CAM importance weight for class *c*
*W*_*j*_	Learnable weights of the residual function at block *j*
𝒵	Perturbed samples generated for LIME
f(·)	Prediction model
g∈G	Interpretable surrogate model for LIME
$\pi_{x}$	Local proximity measure around input *x*
Ω(g)	Complexity penalty for surrogate model

If x is the input, *f*(*x*,*W*_*i*_) represents the series of convolutions and *y* represents the output, then the forward propagation through a residual block can be formulated as per [45]:


$$y=\text{ReLU}\left(f\left(x,\{W_{i}\}\right)+x\right)$$ (1)

The attention layer is placed after the residual blocks to improve spatial awareness and be able to focus on the important regions of the feature maps. Inspired by [46], the custom-made attention layer dynamically assigns different spatial weights to the locations, which allows ARC-Net to highlight the region with a high salience score. The layer has a learnable weight matrix, bias terms, and an attention vector that are initialized with Glorot uniform and zero initializations, respectively. If *A*_*ij*_ is the attention score at spatial location (i,j), *x*_*ij*_ represents the feature vector at that position, *W*_*att*_ is the learnable weight matrix and batt is the bias term, then attention map, A can be calculated as follows:


$$A_{ij}=\frac{\exp(W_{att}\cdot x+b_{att})}{\sum_{i^{\prime }j^{\prime }}\exp(W_{att}\cdot x_{i^{\prime }j^{\prime }}+b_{att})}$$ (2)

Attention scores are computed by applying the softmax operation across the spatial dimensions and then multiplying the result with the feature maps. The attention scores are weighted more towards regions that are more important. Attention layer is then engaged to perform global average pooling thereby reducing the spatial dimensionality, then we apply a fully connected dense layer with ReLU activation to aggregate high-level feature representation. A dropout layer with a 0.5 drop rate is added to prevent overfitting. In the process of minimizing ARC-Net, we use categorical cross-entropy as the loss function and Adam optimizer set to default values with a learning rate to achieve the desired outcomes. The processed features are taken by the classification head and then prepared for the output which also involves a global average pooling layer to reduce dimensions. As described in [47], if fij represents the features at position (i,j) and H and W are the dimensions with a 1D vector z that captures high level info, then:


$$Z=\frac{1}{H\times W}\sum_{i=1}^{H}\sum_{j=1}^{W}f_{ij}$$ (3)

Finally z passes through a fully connected dense layer with ReLU activation with Wout and bout are weights and bias of the dense layer, followed by output layer [48]:


$$\text{Output}=\text{softmax}(W_{\text{out}}\cdot z+b_{\text{out}})$$ (4)

**Fig 3:**
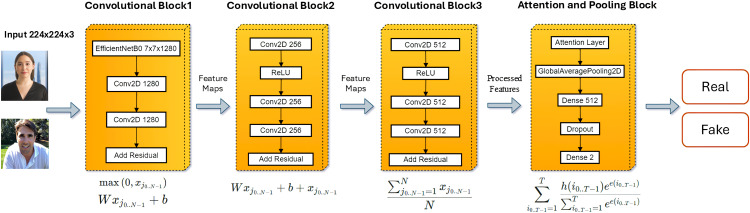
Overall architecture of proposed ARC-Net showing integration of residual and attention blocks atop EfficientNet-B0.

The output layer is a dense layer with two units (binary classification) and a softmax activation that gives class probabilities for “real” and “fake” images. This step further assists the model in differentiating between real and deep fake images. Fig 3 shows the proposed ARC-Net model architecture.

### 3.5 Explainable AI

In computer vision, a technique called Grad-CAM [49] is applied to DL models that are built on Convolutional Neural Networks (CNNs) which helps one understand how CNNs make predictions by providing visual explanations. Heatmaps, known as “Class Activation Maps,” are produced by Grad-CAM. These maps highlight important areas within an image that are accountable for particular CNN predictions. In order to achieve this, it examines gradients that feed into CNN’s last convolutional layer, paying special attention to how these gradients affect class predictions. Guided Grad-CAM produces high resolution detail of the target class in an image by fusing Grad-CAM with pre-existing pixel-space gradient visualizations. Grad-CAM cannot emphasize fine-grained details, despite being class discriminative and able to identify significant image regions. To get around this, high-resolution visualizations are created by fusing the Grad-CAM and Guided Backpropagation images using element-wise multiplication. By negating the gradient of yc with respect to feature maps A of a convolutional layer, one can force the network to alter its predictions. As a result, the important weights are now:


$$\alpha_{k}^{c}=\frac{1}{z}\;\Sigma_{i}\Sigma_{j}-\frac{\partial y^{c}}{\partial A_{i,j}^{k}}$$ (5)

**Fig 4:**
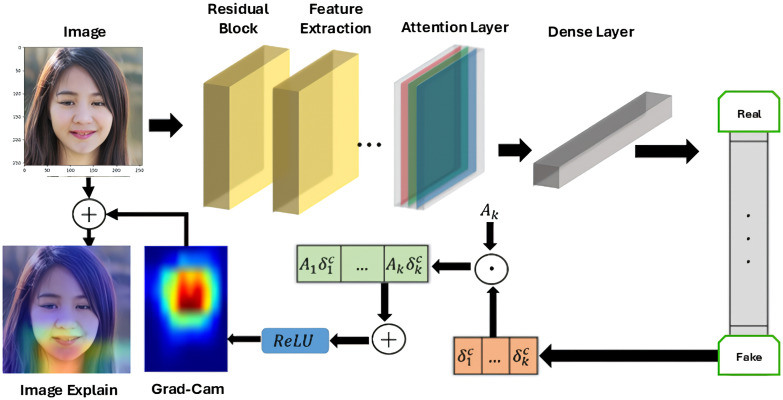
Architecture of the Grad-CAM module used for visualizing attention regions with the ARC-Net model.

Fig 4 shows the Grad-Cam Architecture. Finally, we have also applied LIME like a checkbox in front of a visual image classifier model to understand and present the results. With LIME, we apply superpixels-based changes to the images highlighting the parts that may impact the model’s decision to classify as a DF. LIME focuses on explaining the model’s prediction for specific instances rather than offering a comprehensive knowledge of the model across the entire dataset. The result consists of three primary pieces of information: (1) the model’s predictions; (2) the contributions of the features; and (3) the actual value for each feature. the process of finding a simple, interpretable surrogate model *g* that approximates the complex model *f* locally around a point of interest, weighted by πκ which emphasizes locality, then a formal representation of the optimization objective used in LIME [50]:


$$\epsilon (x)=\arg\min L\left(f,g,\pi_{x}\right)+\Omega (g)$$ (6)

### 3.6 Experimental setup

- For the hardware part, we used 16 GB of DDR5 RAM, an NVIDIA GeForce RTX 4050 graphics card (6 GB DDR6), a 1 TB M.2 NVMe SSD, and an Intel Core i5-13500HX CPU. GPU acceleration was enabled for all deep learning experiments. A full training run of ARC-Net for 30 epochs on our custom “140k Real and Fake Faces and 500 Bangladeshi faces” dataset with the RTX 4050 GPU. This took approximately 2.5–3 hours of wall-clock time (roughly 5–6 minutes per epoch), with peak GPU memory usage remaining below 5 GB as reported by nvidia-smi. Corresponding to the same conditions, per-epoch training times and peak GPU memory usage for the baseline models such as DenseNet121, ResNet50, InceptionV3 and MobileNetV2 were in a similar range, with InceptionV3 being somewhat slower and MobileNetV2 relatively more lightweight (lower peak usage and shorter per-epoch times) that both were at least partially expected. The “Deepfake Dataset” has almost the same number of samples as our benchmark custom dataset, so per-epoch training times are somewhat similar, whereas training on the smaller dataset “Deepfake Database” is significantly faster overall.
- For the software part, we used the Windows Operating System, Python, Tensorflow, Keras API and Jupyter Notebook (Executed with Local CPU/GPU support).

## 4 Result analysis and discussion

The performance of the proposed ARC-Net model is evaluated and compared against four baseline deep learning architectures, namely DenseNet121, ResNet-50, InceptionV3, and MobileNetV2. During training, ARC-Net exhibited strong performance on the validation set without signs of overfitting. The accuracy on the test data further confirms that the model was effectively trained, demonstrating robust generalization beyond the training set. To further assess generalizability, ARC-Net was also evaluated on two additional datasets. Moreover, statistical analyses were conducted to examine the impact of incorporating South Asian (Bangladeshi) images and out-of-distribution (OOD) samples. Finally, the results of the ablation studies and the XAI analysis are presented in this section.

### 4.1 Overall result in 140k real and fake faces dataset

**Fig 5:**
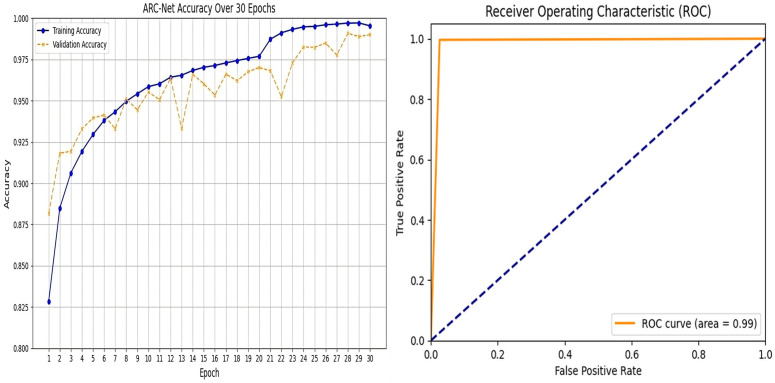
ARC-Net performance analysis. (a) Training/validation accuracy over 30 epochs, and (b) ROC curve showing high AUC score on the ‘140k Real and Fake Faces’ dataset.

**Fig 6:**
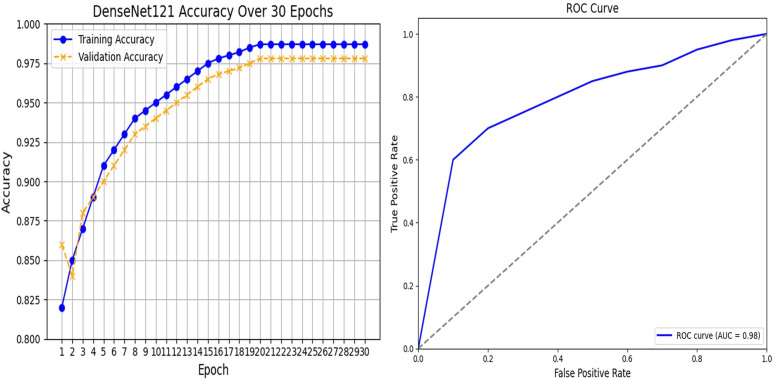
DenseNet121 performance analysis. (a) Training/validation accuracy over 30 epochs, and (b) ROC curve on the ‘140k Real and Fake Faces’ dataset.

**Fig 7:**
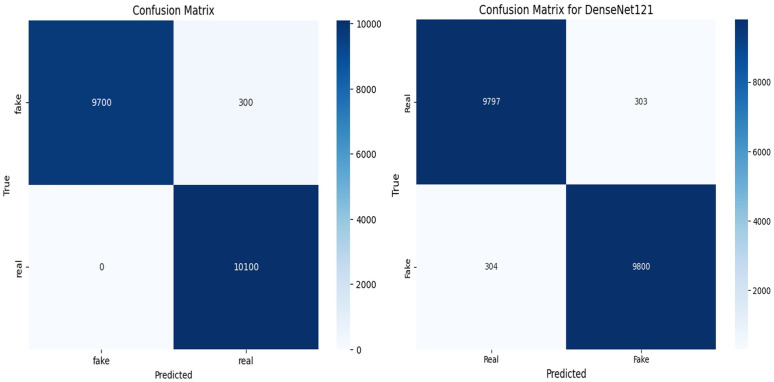
Confusion matrices illustrating the classification performance of ARC-Net. (a) and DenseNet121 (b) on the ‘140k Real and Fake Faces’ dataset.

ARC-Net obtained an accuracy of 99% on a test dataset that contained 20,100 real and fake images. For the training, validation, and test datasets, the accuracy was around 99%. Beyond accuracy, we further validate the model performance through precision, recall, and F1 score evaluation metrics. We also evaluated the confusion matrix to give more insights into the model’s classification ability. The results, with several evaluation metrics, are shown in Table 5 and Table 6 respectively. In Table 5, ARC-Net’s training accuracy of 99.53% and test accuracy of 99% indicate that they generalize well to unseen data. This model also yields the lowest test losses at 0.05, lower than baseline models such as DenseNet121 (0.06), ResNet-50 (0.12), InceptionV3 (0.45), and MobileNetV2 (0.58). The results show that the ARC-Net model converges in training with minimum overfitting, a well-known drawback of DL based models. The classification performance of ARC-Net, presented in Table 6, highlights its effectiveness in distinguishing real and fake images. On real image classification, ARC-Net has 97% precision, 100% recall, and a 0.99 F1 score. For fake images, it also gives 100% precision, 97% recall, and an F1 score of 0.98. These results highlight that ARC-Net is consistently capable of reducing both false positives and false negatives, which is a critically important scalability feature for reliable cross-dataset DF detection. Its balanced precision and recall for both classes show that the model generalizes well for different types of data inputs and does not favor one class of real or fake. The almost perfect classification metrics through ARC-Net demonstrate its ability to learn features at a micro-level, which are often characteristic of DF manipulation. Particularly, the introduction of the attention mechanism in the model architecture is an important reason for this performance, as ARC-Net focuses on those important regions while reducing irrelevant noise. Furthermore, the consistently low-test loss of 0.05 indicates the capability of the model to remain stable on larger datasets, which is necessary in achieving reliable results in real-world applications. ARC-Net shows not only that it is technically superior across a high precision, recall, and F1 score on both real and fake classes but also that it is a potentially strong solution for DF detection. Upon comparison with DenseNet121, ResNet-50, InceptionV3 and MobileNetV2, which served as baseline models, ARC-Net consistently achieved a higher score at all major metrics. DenseNet121 has a test accuracy of 98% with a test loss of 0.06, while ResNet-50, has a lower accuracy of 92% and a higher test loss of 0.12. On the other hand, InceptionV3 and MobileNetV2 have far worse performance, with a test accuracy of 78% and 68% and a test loss of 0.45 and 0.58, respectively which indicates that these models are not able to successfully learn detectors with respect to DF artefacts at subtle levels. Fig 5–6 and Fig 7 show the Train vs. validation accuracy plot, the Receiver Operating Characteristic (ROC) and the confusion matrix for the ARC-Net model and DeseNet121 model.

**Table 5:** Result Evaluation of All Models.

Model	Train Accuracy	Test Accuracy	Test Loss
DenseNet121	98.88%	98%	0.06
ResNet-50	96%	92%	0.12
InceptionV3	79%	78%	0.45
MobileNetV2	70%	68%	0.58
**ARC-Net**	**99.53%**	**99%**	**0.05**

**Table 6:** Classification Report of Models.

Model	Precision		Recall		F1 Score	
	Real	Fake	Real	Fake	Real	Fake
DenseNet121	0.98	0.97	0.97	0.98	0.98	0.98
ResNet-50	0.97	0.98	0.98	0.97	0.97	0.97
InceptionV3	0.89	0.71	0.63	0.93	0.74	0.80
MobileNetV2	0.62	0.81	0.89	0.46	0.73	0.59
**ARC-Net**	**0.97**	**1.00**	**1.00**	**0.97**	**0.99**	**0.98**

ARC-Net is proven to be a reliable solution for DF image detection according to the experimental results. The reason ARC-Net outperforms others is because of its architectural structure: the incorporation of an attention mechanism with its convolutional backbone. The attention mechanism enhances the model’s ability to focus on discriminative regions within the input image, such as facial boundaries, unnatural textures, and lighting inconsistencies, where DF artifacts are most likely to appear. ARC-Net focuses on these important aspects, thus representing subtle and meaningful features that are generally missed by traditional convolutional neural networks (CNNs). This targeted feature extraction results in superior classification performance, as evidenced by its near-perfect precision, recall, and F1 scores. Moreover, the low-test loss of ARC-Net is further confirmation of its reliable optimization and stable convergence. The architecture of the model aims for a good balance between classification of real and DF images accuracy. Moreover, the state-of-the-art performance of ARC-Net is crucial for practical applications of media forensics, cybersecurity, and content verification systems because only the accurate detection of manipulated media can provide assistance to default on its intended purpose.

### 4.2 Generalizability check of ARC-Net

The reliability of the ARC-Net model was assessed through its performance on two benchmark datasets: the deepfake dataset [2] and the deepfake database [3]. Table 7 shows the training and testing accuracy, test loss, precision, recall, and F1-score, which are important measures of how well ARC-Net performed on both datasets.

**Table 7:** Result Evaluation of ARC-Net model on Deepfake Dataset and Deepfake Database.

Dataset	Train Acc. (%)	Test Acc. (%)	Test Loss	Precision (Real/Fake)	Recall (Real/Fake)	F1-Score (Real/Fake)
Deepfake dataset [2]	99.52	97.60	0.0703	0.98 / 0.98	0.98 / 0.98	0.98 / 0.98
Deepfake database [3]	99.87	99.33	0.0231	0.99 / 0.99	0.99 / 0.99	0.99 / 0.99

**Fig 8:**
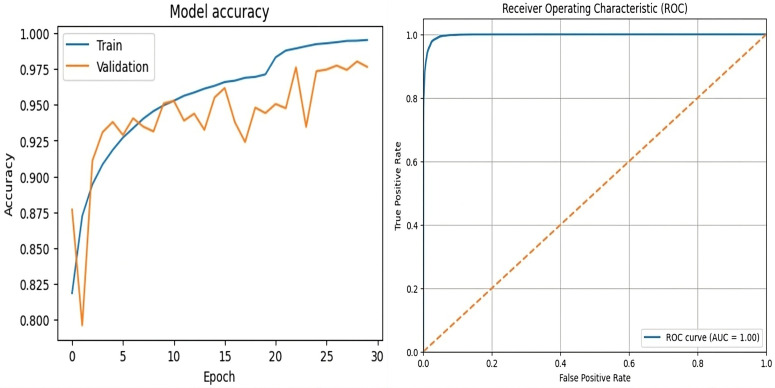
(a) Train validation accuracy plot and (b) Receiver operating characteristic curve of ARC-Net on the Deepfake dataset.

**Fig 9:**
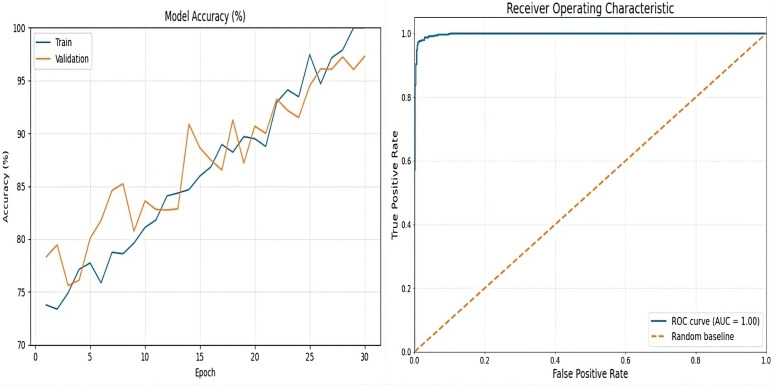
(a) Train validation accuracy plot and (b) Receiver operating characteristic curve of ARC-Net on the deepfake database.

On the deepfake dataset ARC-Net achieved 99.52% training accuracy and 97.60% testing accuracy which resulted in a generalization gap of 1.92%. The test loss of 0.0703 confirms stable convergence without severe overfitting. The precision and recall values reached 0.98 for both real and fake classifications which resulted in F1-scores of 0.98 for each class while maintaining balanced control over false positives and false negatives in a highly diverse sample space. Also, when ARC-Net applied to the smaller deepfake database resulted in outstanding performance with 99.87% training accuracy and 99.33% testing accuracy and a minimal test loss of 0.0231. The precision and recall values achieved 0.99 for both real and fake labels which produced F1-scores of 0.99 for all classes. The high metrics obtained on the both large and small dataset prove that ARC-Net performs well on different dataset sizes and complexity levels. Fig 8 and Fig 9 show the train validation accuracy plot and receiver operating characteristic curve of ARC-Net on the deepfake dataset and deepfake database.

**Fig 10:**
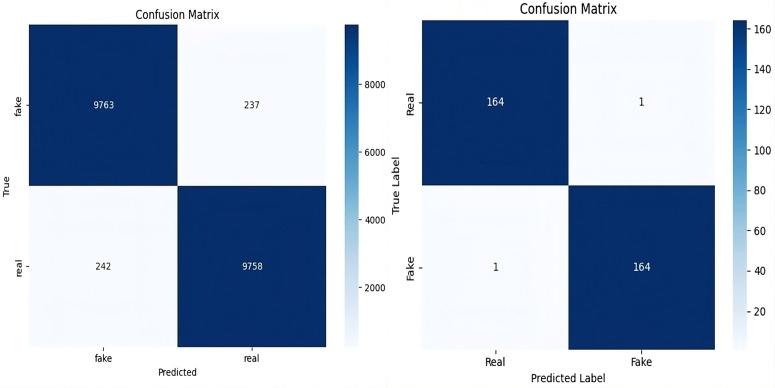
Confusion matrix of ARC-Net in (a) deepfake dataset and (b) deepfake database dataset.

**Fig 11:**
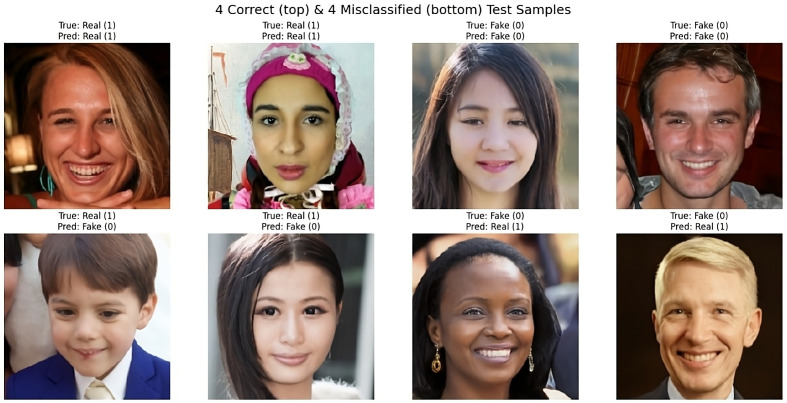
Correct and incorrect test examples from the deepfake dataset with ARC-Net.

Fig 10 shows the confusion matrix of ARC-Net on the deepfake dataset and deepfake database. On Deepfake Dataset, ARC-Net identified 9,763 manipulated images as fake while incorrectly identifying 237 images as real and identified 9,758 authentic images as real but misidentified 242 images as fake. On the Deepfake Database, ARC-Net correctly identified 164 real images but mistakenly identified one real frame as fake and identified 164 manipulated images as fake but incorrectly identified one manipulated images as real. Fig 11 shows four examples from the deepfake dataset test set that were either correctly classified or misclassified by ARC-Net. The top row shows four correctly classified images (two real, two fake), while the bottom row shows four misclassifications (two false positives, two false negatives). Table 8 presents comparison of several research studies on deepFake face detection with our proposed ARC-Net model. The reliability assessment results collectively demonstrate that ARC-Net is robust for real-world deepfake detection and its consistent high performance on both large and small datasets shows its ability to classify data at different scales in a balanced and generalizable way.

**Table 8:** Comparison of classification performance across various research studies on the DeepFake face detection with our proposed ARC-Net model.

Study	Technique	Best Result	Dataset	Explainability	Generalizability
Shahzeb Naeem et al. [21]	ViT Patch-16	Sensitivity, specificity, precision, and accuracy of 97.37%, 98.69%, 97.48%, and 98.25%, respectively.	SFHQ-1, 140k Real and Fake Faces and 1 Million Fake Faces Dataset	×	×
H. S. A. Kareem et al. [17]	Support Vector Machine (SVM) with Principal Component Analysis (PCA)	96.80% of Accuracy	Flickr and Bojan’s 1 Million Dataset	×	×
E. Şafak et al. [23]	Stacking (EfficientNetB0, MobileNet, MobileNetV2)	96.44% of highest accuracy	FFHQ dataset	×	×
Kothandaraman D et al. [24]	InceptionResNetV1	97.00% of highest accuracy	VGGFace2 dataset and Kaggle DeepFake Classification Dataset	×	×
Jatin Sharma et al. [25]	Ensemble approach with VGG16 and ResNet50	Accuracies on the 3 datasets as 98.79%, 75.79%, and 95.52%, respectively.	140k Real and Fake Faces, Real and Fake Face Detection, and Fake Faces dataset	×	×
Mathews et al. [43]	MesoInception-Net	99.87% validation accuracy	DFIM-HQ dataset	Grad-Cam	×
H. Alshammari et al. [27]	MesoNet	96.20% accuracy with sensitivity of 97.48% and a specificity of 94.75%	140k Real and Fake Faces dataset	×	×
B. Sugiantoro [44]	ResNet152V2 + Grad-CAM	Highest Precision of 90% for fake and 92% for real images	FFHQ dataset	Grad-Cam	×
**This Study**	**Proposed ARC-Net**	**99.00%, 97.60% and 99.33% of Accuracy in 3 different datasets**	**140k Real and Fake Faces, Deepfake dataset and Deepfake database**	**Grad-CAM and LIME**	**Cross-dataset validation on Deepfake dataset and Deepfake database.**

### 4.3 Impact of incorporating South Asian data and OOD generalization

This section evaluates the impact of incorporating a small set of South Asian (Bangladeshi) images into the training and validation datasets to analyze its impact on model performance. Additionally, the out-of-distribution generalization of ARC-Net is examined through two experiments involving multiple unseen deepfake datasets.

#### 4.3.1 Impact of South Asian (Bangladesh) images.

An extensive experiment was conducted to evaluate the impact of incorporating 400 real South Asia (Bangladesh) images into model training and validation on subgroup performance. The models were evaluated on a held-out set of 100 real South Asia (Bangladesh) images. Evaluation metrics included the False Positive Rate (FPR: real → fake), Mean Probability of Real (Mean prob_real), Median Probability of Real (Median prob_real), Brier Score, and McNemar’s paired test statistic. Table 9 provides detailed model definitions, while Table 10 compares the performance of Model A and Model B on the held-out South Asia (Bangladesh) test set.

**Table 9:** Model Configurations and Experimental Setup.

Experiment ID	Training set composition	Validation set	Purpose
Model A – ARC-Net (no South Asian images)	100,000 images (original corpus), excluding South Asia (Bangladesh) images	20,000 images (original validation set), excluding South Asia (Bangladesh) images	Measure out-of-domain (OOD) performance when no in-domain South Asia (Bangladesh) samples are present
Model B – ARC-Net (with South Asian images)	100,000 original images + 200 South Asia (Bangladesh) real images	20,000 original validation images + 200 South Asia (Bangladesh) real images	Measure the effect of adding a small, targeted set of in-domain Bangladeshi real images on subgroup performance.

**Table 10:** Comparison of Model A and Model B on the Held-Out South Asian Test Set (100 Real Images).

Metric	Model A – ARC-Net (no South Asian images)	Model B – ARC-Net (with South Asian images)
FPR (%)	22.0 [14.3–31.4]	10.0 [4.9–18.0]
Mean prob_real	0.7200	0.8600
Median prob_real	0.7300	0.7300
Brier Score	0.1800	0.1100
McNemar paired test		p = 0.028 (significant improvement for Model B)

**Fig 12:**
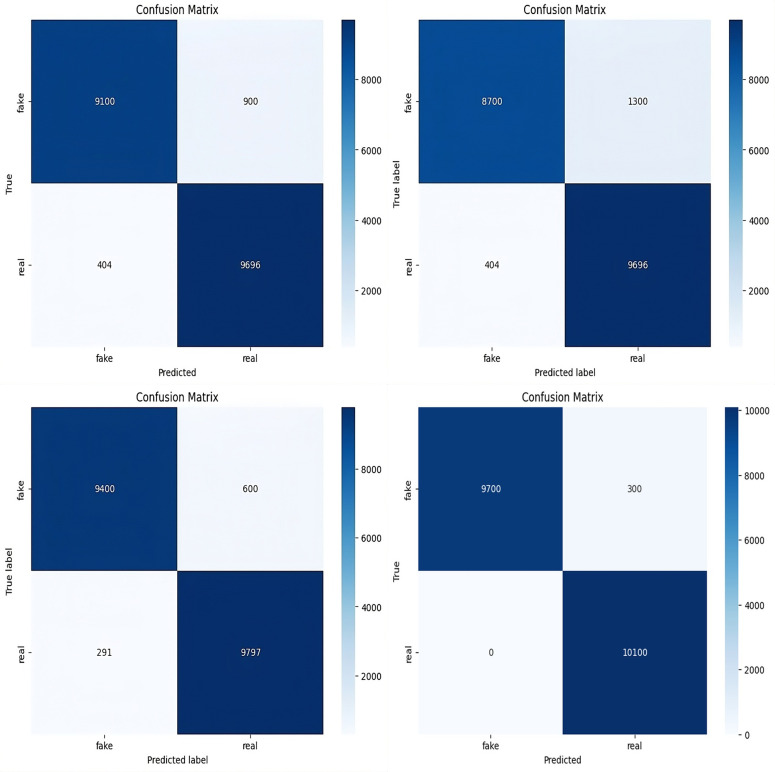
False Positive Rate change and McNemar contingency table between Model A and Model B.

**Fig 13:**
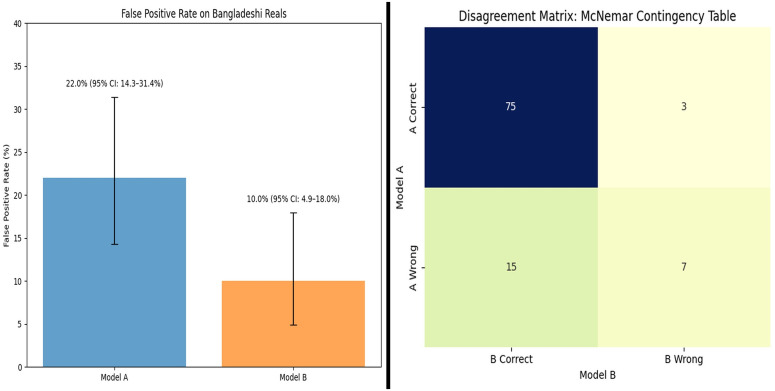
Confidence distributions of mean probability of real for Model A (blue) and Model B (orange).

The FRP analysis demonstrates that using a limited number of real South Asian (Bangladeshi) images from the in-domain dataset leads to better results on the South Asian held-out set. The addition of less than 1% of the training data resulted in significant performance enhancements for the corresponding subgroup. Model A, without South Asian images in training and validation, exhibited a false positive rate (FPR) of 22.0% (95% CI: 14.3–31.4), and Model B, which had the additional 400 South Asian images included in training and validation, shows an FPR of 10.0% (95% CI: 4.9–18.0). The inclusion of 400 South Asian images in Model B resulted in a 12 percentage point absolute FPR decrease and a 54.5% relative FPR reduction (shown in Fig 12). On the other hand, the McNemar contingency table shows paired classification results from the 100-image test set where both models achieved correct results in 75 cases, Model A was correct while Model B was incorrect in 3 cases, Model A was incorrect while Model B was correct in 15 cases, and both models failed in 7 cases. Model B improved Model A’s accuracy by fixing 15 of its wrong predictions, but Model A only corrected 3 of Model B’s incorrect predictions. The McNemar test produced a p-value of 0.028, which shows Model B outperformed Model A at a statistically significant level (alpha = 0.05) (Fig 12). The results from calibration analysis confirm the improvement in the model. Model A’s Brier score is 0.180, but Model B performed better with a Brier score of 0.110, which demonstrates improved probability calibration for Model B when predicting South Asian (Bangladesh) images. Model B demonstrates better alignment between its predicted probabilities and actual results, which decreases the probability of making overly confident or underconfident predictions on South Asian (Bangladesh) images. Fig 13 presents the distribution of predicted confidence scores, further supporting the observed reduction in error rates. For the “real” class, Model A’s predicted probabilities had a mean of 0.72 and a median of 0.73, indicating that the model assigned much of its probability mass near the decision boundary and exhibited relatively high uncertainty. In contrast, Model B demonstrated a notable increase in confidence, with a mean predicted probability of 0.86 for the same class, while the median remained at 0.73. This suggests that Model B became more confident in its predictions for authentic images, contributing to its improved performance. The results from error-rate, confidence distribution, paired-comparison and calibration tests show that adding less than 1% of Bangladeshi real images to the training set leads to significant reduction of subgroup bias. The addition of 400 South Asian (Bangladesh) real images to training and validation data sets resulted in a more than 50% decrease in false positive rates and better probability calibration which shows that focused interventions with limited data can improve detection accuracy for minority groups.

#### 4.3.2 Out-of-distribution evaluation 01 (OOD-1).

The first out-of-distribution evaluation (OOD-1) trained ARC-Net using 140,000 real and fake face images from the 140k Real/Fake Faces dataset (Dataset A). The model demonstrated its generalization capabilities through testing on two separate datasets including the Deepfake Database (Dataset B) and the Deepfake Dataset (Dataset C) which it had not encountered during training. The evaluation tests how well ARC-Net performs on data points that exist beyond its training data range. Table 11 shows the dataset distribution and results of OOD Evaluation 01.

**Table 11:** Dataset Distribution and Results of OOD Evaluation 01.

Dataset	Description	Accuracy	Precision	Recall	F1-score
Dataset A	140k Real and Fake Faces	98.5%	0.98	0.97	0.98
Dataset B	Deepfake Database	94.2%	0.94	0.94	0.94
Dataset C	Deepfake Dataset	93.7%	0.94	0.94	0.94

The proposed ARC-Net model which uses EfficientNetB0 backbone with attention and residual mechanisms achieved high performance in both in-domain and out-of-distribution (OOD) testing according to Table 11. The model achieved 98.5% validation accuracy after training on Dataset A which contained 140k Real and Fake Faces images. The model underwent evaluation for generalization performance on two new datasets which were not used during training. The model achieved 94.2% accuracy on Dataset B (Deepfake Database) while maintaining equal precision and recall and F1-score values of 0.94 for both fake and real classes. The model achieved 93.7% accuracy on Dataset C (Deepfake Dataset) while maintaining F1-scores of 0.94 for each class.

**Fig 14:**
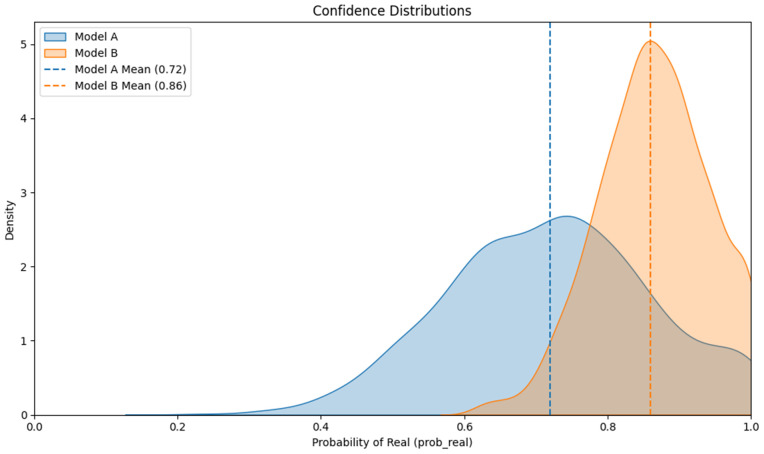
Performance comparison of OOD-01.

The results show that ARC-Net achieves successful generalization to new datasets which contain different source materials and deepfake production methods (shown in Fig 14). The model demonstrates strong real-world application potential through its consistent high performance and balanced results on both Dataset B and Dataset C under distributional shift conditions.

#### 4.3.3 Out-of-distribution evaluation 02 (OOD-2).

For this OOD-2 experiment, the model was trained solely on Dataset A (Deepfake Dataset) and tested on two distinct external datasets: Dataset B (Deepfake Database) and Dataset C (140k Real and Fake Faces). Table 12 shows the dataset distribution and results of OOD Evaluation 02.

**Table 12:** Dataset Distribution and Results of OOD Evaluation 02.

Dataset	Description	Accuracy	Precision	Recall	F1-score
Dataset A	Deepfake Dataset	0.98	0.97	0.98	0.98
Dataset B	Deepfake Database	0.94	0.95	0.93	0.94
Dataset C	140k Real and Fake Faces	0.92	0.93	0.91	0.92

**Fig 15:**
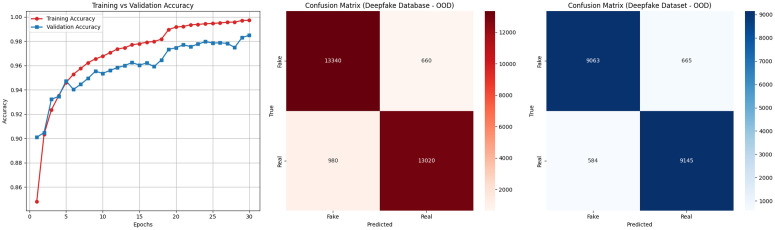
Performance comparison of OOD-02.

The OOD-2 evaluation process used the Deepfake Dataset (Dataset A) for training ARC-Net while validating with 20% of the data. The model achieved 98% training and validation accuracy when tested on the same dataset because it learned powerful discriminative features. The trained model received direct application to two new datasets which included the Deepfake Database (Dataset B) and the 140k Real and Fake Faces dataset (Dataset C). The trained model achieved 94% accuracy on Dataset B while maintaining balanced precision–recall performance for real and fake class detection. The model achieved 92% accuracy when applied to Dataset C although its performance decreased minimally from Dataset B results. Fig 15 shows the Performance comparison of OOD-02.

The results demonstrate ARC-Net’s ability to handle distribution shifts because the model successfully applies learned representations to new data regardless of source differences and generation methods and data quality variations. The results show ARC-Net operates beyond single-dataset limitations because it demonstrates effective out-of-distribution generalization.

### 4.4 Ablation studies

We performed controlled experiments to evaluate the individual impact of architectural components in ARC-Net by removing or isolating the residual and attention modules. The ablation variants received the same training and evaluation data as the full model while undergoing identical optimization procedures until validation set convergence. Table 13 lists the architectural configurations for each experiment and Table 14 presents the performance metrics for each variant.

**Table 13:** Experiment Configurations.

Experiment ID	Architecture Description
A	EfficientNetB0 (frozen) → GlobalAveragePooling2D → Fully-Connected layers
B	A + AttentionLayer inserted just before GlobalAveragePooling2D
C	A + Stack of residual blocks inserted before GlobalAveragePooling2D
D	DenseNet121 (frozen) → ResidualBlock stack → AttentionLayer → GlobalAveragePooling2D → Dense layer(s)
Our Proposed	EfficientNetB0 (frozen) → ResidualBlock stack → AttentionLayer → GlobalAveragePooling2D → Dense layer(s)

**Table 14:** Performance of ablation variants and the full ARC-Net model on the hybrid dataset.

Experiment ID	Accuracy (%)	Test Loss	Precision (R/F)	Recall (R/F)	F1-Score (R/F)
A	94.00	0.1607	0.92 / 0.96	0.96 / 0.91	0.94 / 0.94
B	92.00	0.2265	0.88 / 0.96	0.92 / 0.87	0.92 / 0.91
C	95.00	0.1294	0.94 / 0.97	0.97 / 0.94	0.95 / 0.95
D	97.50	0.0850	0.94 / 0.97	0.97 / 0.93	0.95 / 0.95
Our Proposed	99.00	0.0500	0.97 / 1.00	1.00 / 0.97	0.99 / 0.98

The frozen EfficientNetB0 backbone with GAP and FC classifier reached 94% accuracy and 0.1607 loss in Experiment A (baseline). The pretrained feature extractor demonstrates its base capability through this result without any additional modules. The addition of the attention module in Experiment B led to a minor reduction in total accuracy to 92% and a higher loss value of 0.2265. The attention mechanism enhanced the precision of fake class detection to 0.96 but simultaneously reduced the recall of fake samples to 0.87 which indicates that standalone attention might prioritize specific spatial features over overall robustness. Experiment C achieved 95% accuracy with a 0.1294 loss when residual blocks were added before GAP. The residual-only variant enhanced balanced feature learning because precision and recall metrics exceeded 0.94 for both classes. The backbone of Experiment D used DenseNet121 which received additional functionality through residual and attention modules. The combination of DenseNet with residual and attention modules produced 97.5% accuracy because it leveraged the strong feature propagation abilities of DenseNet for balanced precision and recall performance. The independent model evaluation in Table 03 showed that DenseNet121 reached 98% test accuracy while maintaining uniform performance across all classes and achieving equal precision and recall values. The standalone model of EfficientNetB0 reached 94% accuracy but demonstrated less balanced precision and recall performance compared to the results. The results indicate that DenseNet121 performs better than EfficientNetB0 when operating independently.

The comprehensive ARC-Net configuration, incorporating residual blocks prior to the attention layer, demonstrated superior performance, attaining 99% accuracy, a test loss of 0.05, and near-perfect F1-scores, thereby outperforming both the baseline models and the DenseNet121-based hybrid architecture. This gain can be attributed to the architectural compatibility between EfficientNetB0 and the residual-attention modules. While DenseNet121 inherently promotes dense feature reuse, this may diminish the added value of external residual blocks and introduce feature redundancy. In contrast, EfficientNetB0 provides a more compact and computationally efficient representational space, enabling residual and attention mechanisms to refine discriminative features more effectively and capture long-range dependencies. The combination of residual and attention modules produced perfect recall on real images and perfect precision on fake images, indicating strong discrimination capability.

**Fig 16:**
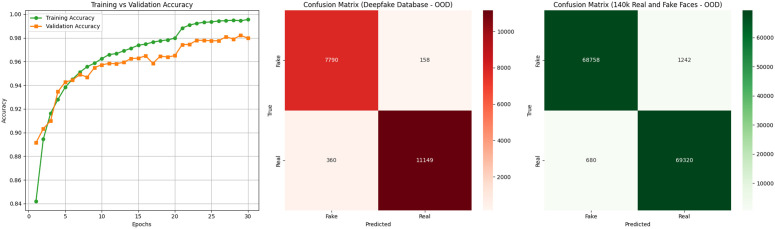
Confusion Matrix of ARC-Net analysis: **(a)** Baseline EffecientNetB0 (Experiment **A)**, **(b)** Attention bassed CNN (Experiment B) and **(c)** Residual CNN (Experiment C)in hybrid dataset. **(d)** DenseNet121-based CNN with residual and attention modules (Experiment **D)**.

The ablation results demonstrate that residual blocks and attention modules work synergistically: residual connections preserve feature propagation and stability, while attention mechanisms focus on subtle artifacts. This integration yields a significant accuracy improvement of +5.0% over the baseline (A), + 4.0% over the top single-module variant (C), and +1.5% over the DenseNet121-based hybrid (D), validating the complementary role of these modules in enhancing ARC-Net’s performance. The confusion matrices for Experiments A, B, C and D are presented in Fig 16.

The ablation results demonstrate that both residual blocks and the attention mechanism play crucial roles in ARC-Net’s architecture because residual blocks enable deep feature extraction without loss of information and the attention mechanism enhances the detection of forgery cues.

### 4.5 Explainability with Grad-Cam & Lime

**Fig 17:**
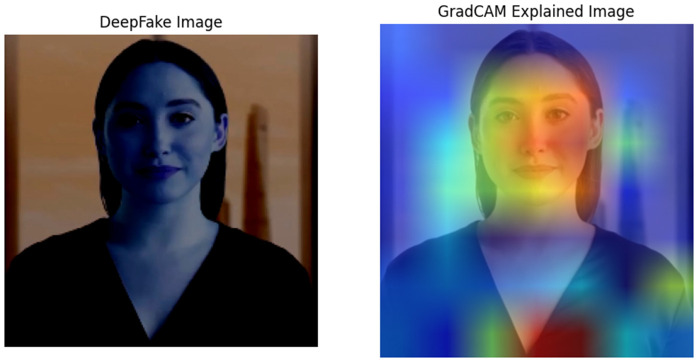
Grad-CAM Explanation and Heatmap with ARC-Net.

**Fig 18:**
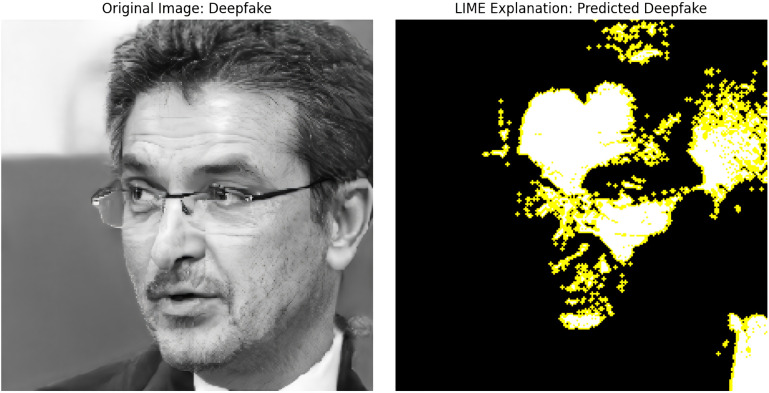
LIME Explanation for ARC-Net.

From our study, we need to ensure that, although our proposed ARC-Net performs well, the model’s decision-making process must be explainable. This is important to verify that our approach is correctly identifying the regions of a facial image to detect DF or Real images, which is crucial for the model’s reliability and trustworthiness. We applied the Grad-CAM and LIME XAI techniques to the ARC-Net model. To illustrate both XAI techniques, we examined a random image from our dataset, as shown in Fig 17 and Fig 18 respectively.

Grad-CAM displays the portion of the input image that contributes the most to the development of ARC-Net classification. ARC-Net predicts the image to DF, demonstrating the subtlety of DF artifacts in the input, as shown in Fig 17. As visualized through the Grad-CAM heatmap, ARC-Net attends mainly to facial features, which include the eyes, nose, and mouth, and their boundaries peripheral to the face. The heatmap also detects texture mismatches and light discrepancies, which are also common signatures of DF alterations. This phenomenon is especially relevant in high-detail areas where injected synthetic content tends to create artifacts. Aligning ARC-Net with this targeted focus, its architectural design makes use of an attention mechanism for augmenting the learning of discriminative features. ARC-Net effectively reduces misclassification particularly where DF artifacts are fine-grained and local in nature by isolating and focusing attention only over high impact regions.

**Fig 19:**
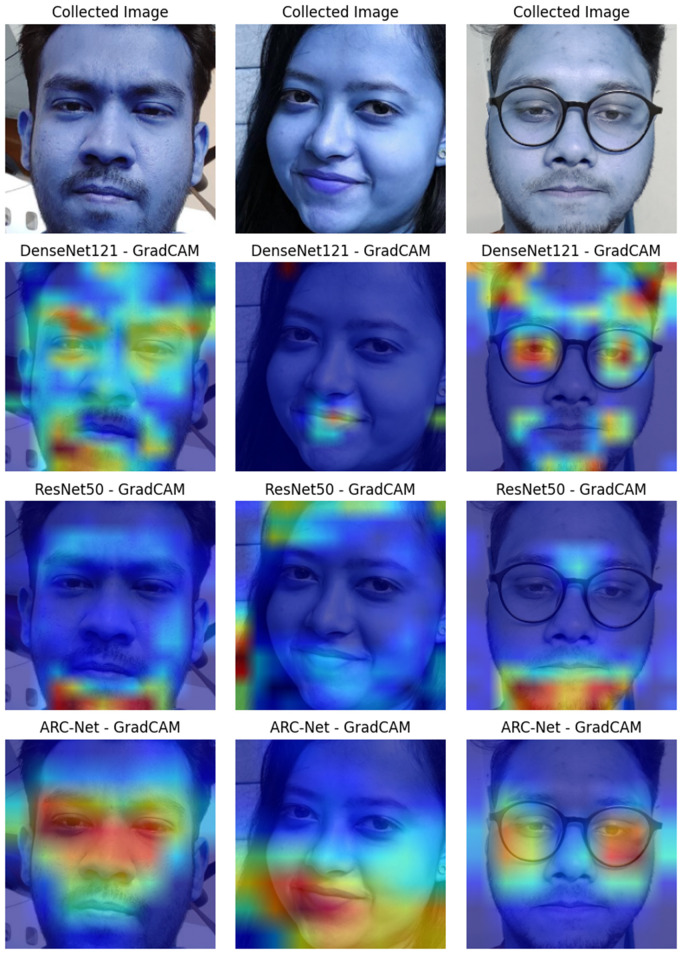
Grad-CAM Explanation and Heatmap for DenseNet121, ResNet50, and ARC-Net on real human faces.

The way LIME works is by perturbing the input features (pixels) and checking the response of the model outputs; it then finds the regions that influence the classification most. As an example, Fig 18 is a DF image, and LIME explanation of the image, with the yellow parts being the most essential parts of the image in making the prediction. As shown in the LIME output, ARC-Net mainly concentrates on texture regions such as the forehead, eyes, and mouth. Parts of these regions can create artifacts due to how pixel blending works on a DF, lighting irregularities, and misaligned facial structures. The LIME Map confirms that ARC-Net learnt to rely on these yellow-highlighted regions as critical regions for making its decision of classifying. Moreover, to evaluate the performance of the ARC-Net, we compared the performance of DenseNet121 and ResNet50 by taking three human real facial images from the testing data as shown in Grad-CAM visualisations in Fig 19 to see which regions of the image each model focuses on. The attention of ARC-Net is highly accurate and focused only on the most important face regions, such as the eyes, mouth, lips and contours that are strongly indicative of DF artefacts. On the other hand, DenseNet121 demonstrates slightly better focus compared to ResNet50 but still lacks the sharp, targeted attention achieved by ARC-Net. ResNet50 is less reliable for real or DF detection because it exhibits noisy and scattered activations, frequently highlighting irrelevant regions.

Both Grad-CAM and LIME confirm that the model’s predictions are based on relevant and discriminative features, rather than spurious correlations or background noise. By using Grad-CAM and LIME, we make ARC-Net’s outputs explainable by highlighting areas that are associated with DF artifacts like facial textures, lighting anomalies or edge distortions. These methods aid in enhancing the transparency and reliability of ARC-Net’s predictions, thereby enhancing its usefulness in various scenarios such as media forensics, cybersecurity, and content verification systems. Grad-CAM indicates that ARC-Net pays attention to facial areas and texture inconsistencies, while LIME indicates such sensitivity to high-texture regions susceptible to DF artifacts. This alignment validates the model’s decisions, reinforces its robustness, and positions ARC-Net as a trustworthy solution for real-world DF detection.

### 4.6 Quantitative evaluation of explainability localization

We performed a quantitative interpretability analysis on a manually annotated representative subset of the data (N = 200, 100 fake and 100 real) to supplement the qualitative Grad-CAM explainable AI visualizations. In order to ensure transparency and reproducibility, annotations were produced using the VGG Image Annotator (VIA) and converted into binary masks resized to 224×224. Following a split 70−30 ratio, the dataset of 200 manually annotated facial images was used for training and testing where train and test set (140 training images and 60 held-out test images) were used to evaluate XAI localization performance. Our proposed ARC-Net model is an attention-residual aware and spatially oriented convolutional neural network that integrates advanced residual connections with channel-spatial attention modules. It uses an EfficientNetB0 backbone as its base feature extractor enabling efficient transfer learning while maintaining strong generalization capacity under limited data regimes. The heatmaps produced by GradCAM were thresholded and converted into binary relevance masks which were then compared with the expert human-annotated reference masks. A consistently high localization precision was achieved with a mean Intersection-over-Union (IoU) of 0.8396 (95% CI: 0.8071–0.8684) and a mean Dice similarity coefficient of 0.9121 (95% CI: 0.8931–0.9284), as shown in Table 15. Moreover 88.9% of all test samples exhibited peak activation within the annotated region of interest, which suggests that the ARC-Net attention and feature-refinement modules effectively guide the network to salient facial areas relevant for real vs deepfake. The models stability was further confirmed by a per image analysis with IoU values ranging from 0.7492 to 0.8937 and Dice scores ranging from 0.8566 to 0.9438 where ARC-Net not only performs accurate classification but also provides spatially coherent and semantically aligned classification.

**Table 15:** Representative IoU, Dice, and Peak-Activation Scores for a Subset of Test Images. The complete dataset comprises 200 images; only a concise selection is included here for clarity. Aggregate metrics (mean and 95% CI) are computed over the full test set.

Image ID	IoU	Dice	Peak-in-Mask
T-01	0.7492	0.8566	1
T-02	0.8326	0.9086	1
T-03	0.8426	0.9146	0
T-04	0.7904	0.8829	1
T-05	0.8937	0.9438	1
T-06	0.8664	0.9284	1
T-07	0.8103	0.8952	1
T-08	0.8890	0.9412	1
T-09	0.8824	0.9375	1

## 5 Conclusion and future work

The emergence of DF, and more specifically, look like realistic facial content but fake image, is creating a serious threat to media forensics, cybersecurity, and content authentication. Previous studies have utilized extensive research; however, it remains unclear whether their models can accurately focus on deepfake facial regions or if their approaches are trustworthy. Moreover, most of the datasets used predominantly represent groups outside South Asia. To address this gap, the present study constructed a hybrid dataset by combining a publicly available dataset of 140K real and fake faces with 500 images of individuals from Bangladesh, thereby enhancing the dataset’s diversity. We present ARC-Net (Attention Residual Convolutional Network with EfficientNetB0 Base), an innovative approach CNN Architecture for DF facial image detection. Our proposed architecture, ARC-Net, gives a 99% test accuracy and achieves state-of-the-art results on DF human face detection compared to conventional pre-trained DL models using DenseNet121, MobileNetV2, ResNet50, and InceptionV3. Additionally, ARC-Net was evaluated on both the deepfake dataset and the deepfake database to assess its generalizability, achieving strong performance on the former and 97.60% and 99.33% accuracy respectively. Incorporating South Asian (Bangladesh) images into the training and validation sets, representing fewer than one percent of the total training data, reduced the false positive rate from 22.0% to 10.0% and improved the Brier score from 0.180 to 0.110, indicating more accurate and confident predictions for this underrepresented subgroup. Further out-of-distribution (OOD) evaluations demonstrated that ARC-Net generalized effectively to unseen datasets, achieving 94.2% and 93.7% accuracy in the first OOD experiment, and 94% and 92% accuracy in the second OOD experiment, with balanced precision, recall, and F1-scores across all datasets. Ablation studies revealed that the combination of residual and attention modules increased accuracy by 5.0% over the baseline EfficientNetB0, 4.0% over the top single-module variant, and 1.5% over a DenseNet121-based hybrid, highlighting the complementary role of these modules in enhancing feature discrimination and model performance.

ARC-Net not only achieves higher accuracy but also has the ability to fully capture nuances unique to manipulation, as it focuses on and gives attention to the facial region, where traditional DL models may fall short. Grad-CAM and LIME further confirmed the interpretability of the model by visualizing the discriminative regions it focused on, such as facial textures, lighting anomalies, and edge distortions, thereby confirming the reliability and explainability of the predictions.

Overall, ARC-Net offers a strong, explainable, geographically inclusive, and geographical-wide DF facial image detection approach, overcoming some of the major drawbacks of existing methods while achieving comparable or even better performance in almost all settings. Its contributions represent an important foundation for future progress in addressing the dynamic problem of DF generation, rendering it as a scalable and trustworthy solution for real-world deployments in media forensics, cybersecurity, and content verification systems.
